# The general practice specialist examination – examiners’ perspectives on the status quo and future development: An exploratory qualitative study

**DOI:** 10.3205/zma001872

**Published:** 2026-09-15

**Authors:** Andreas Christian Dreher, Christine Becker, Marco Roos, Attila Altiner, Simon Schwill

**Affiliations:** 1 University Hospital Heidelberg, Department for Primary Care and Health Services Research, Heidelberg, Germany; 2 University of Augsburg, Faculty of Medicine, Department of General Practice, Augsburg, Germany

**Keywords:** specialist examination, examiner training, postgraduate medical education, Competence Centres for Postgraduate Training, general practice

## Abstract

**Background:**

The specialist examination has a substantial impact on the quality of healthcare delivery. The aim of this study was to explore examiners’ perspectives on the current examination format, their motivation for serving as examiners, perceived strengths and weaknesses of the existing assessment, suggestions for improvement, and the perceived effects of Competence Centres for Postgraduate Training (CCPTs).

**Methods:**

Examiners from across Germany were recruited and interviewed in semi-structured video interviews using a self-developed interview guide. Interviews were transcribed verbatim and analysed using qualitative content analysis.

**Results:**

Twelve examiners from six federal states participated. The mean interview duration was 33 minutes (range: 24-57 minutes). Key advantages identified included the one-to-one examination setting, the ability to ensure a minimum standard of specialist competence and the examination's efficiency in terms of time and resources. Oral examinations were perceived as having limited ability to assess the complex clinical competencies required in patient care. Perceived needs for change varied among participants. Suggested improvements included examiner training, the development of shared assessment criteria and the introduction of practical examination components. Longitudinal assessments were also mentioned.

**Discussion:**

Examiners recognised limitations of the current examination regarding objectivity, reliability, validity and contextual sensitivity, particularly in relation to the complexity of general practice. Nevertheless, they considered the assessment of a minimum standard of specialist competence to be feasible. The findings reveal a tension between practical feasibility and educational assessment standards. Potential areas for improvement include examiner training, regular exchange among examiners, shared assessment frameworks, a clearer definition of examination objectives and the implementation of longitudinal assessment approaches (e.g. portfolio-based assessment with regular interim assessments).

Conclusions: The specialist examination demonstrates considerable potential for further development. Shared assessment frameworks, as developed during examiner training, could provide greater structure to the examination process and may be implemented with relative ease.

## Zusammenfassung

**Hintergrund:**

Die Facharztprüfung beeinflusst in hohem Maße die Versorgungsqualität. Ziel der Studie war die Perspektiven von Prüfenden zu Status quo, Motivation, Vor- und Nachteilen, Veränderungsvorschlägen sowie Effekten der Kompetenzzentren Weiterbildung (KW) zu explorieren.

**Material und Methoden:**

Prüfende wurden deutschlandweit rekrutiert und anhand eines selbstentwickelten Fragebogens in semi-strukturierten Video-Interviews befragt. Die Aussagen wurden Wort für Wort transkribiert und inhaltanalytisch ausgewertet.

**Ergebnisse:**

Es nahmen 12 Prüfende aus 6 Bundesländern teil. Die durchschnittliche Interviewdauer betrug 33 Minuten (Min. 24 min, Max. 57 min). Als Vorteile wurde das Einzelprüfungssetting, die Sicherung des Mindeststandards sowie die Zeit- und Kosteneffizienz hervorgehoben. Als nachteilig wurde die begrenzte Aussagekraft mündlicher Prüfungen für die komplexen Handlungskompetenzen an Patient:innen gesehen. Der wahrgenommene Veränderungsbedarf variierte zwischen den Prüfenden. Vorgeschlagen wurden Schulungen, Erwartungshorizonte und praktische Prüfungsanteile. Zudem wurden longitudinale Assessments benannt.

**Diskussion:**

Prüfende sehen Schwächen der Prüfung bezüglich Objektivität, Reliabilität, Validität und Kontextsensibilität in Bezug auf die Komplexität allgemeinmedizinischer Arbeitsweise, halten aber die Einschätzung des Mindeststandards der Facharztqualifikation für möglich. Es zeigt sich ein Spannungsfeld zwischen praktischer Umsetzbarkeit und prüfungsdidaktischem Anspruch. Potenziale zur Weiterentwicklung bestehen in Prüfendenschulungen, regelmäßigem Austausch, gemeinsamen Erwartungshorizonten, einer geschärften Zieldefinition und longitudinalem Assessment (Portfolio mit regelmäßigen Teilprüfungen).

Schlussfolgerung: Die Facharztprüfung weist Entwicklungspotenzial auf. Vergleichbare Erwartungshorizonte, wie sie bei Prüfendenschulungen erlernt werden, strukturieren die Prüfung und könnten einfach implementiert werden.

## Introduction

The specialist examination in general practice marks the completion of five years of full-time equivalent postgraduate training [1]. In Germany, the German medical association is responsible for administering these examinations, which serve as the formal certification of specialist competence. As the final assessment in a physician’s professional training pathway, successful completion permits independent and autonomous (specialist) practice. Consequently, the specialist examination represents an important instrument for ensuring specialist qualification, with direct implications for healthcare quality and patient safety [2], [3]. The examination usually takes the form of an oral assessment lasting at least 30 minutes and is conducted by two specialist examiners and a chairperson. Traditionally, it consists of an unstructured collegial oral discussion, a format that has been firmly established in Germany for decades. However, its implementation may vary according to region, specialty and individual examination practices.

The introduction of a five-year postgraduate training programme and a specialist examination in general practice represented a major step towards achieving parity between general practice and other medical specialties. In recent years, the number of specialist examinations in general practice has increased considerably [4]. In Baden-Württemberg (federal state of Germany), for example, the examination currently constitutes the most frequently administered specialist examination [5]. Failure rates in German specialist examinations are generally in the single-digit percentage range, whereas the European specialist examination in emergency medicine reports failure rates of up to 64% [6]. In addition to workplace-based training under supervision, Competence Centres for Postgraduate Training (CCPTs) have been established in Germany to enhance the quality and efficiency of postgraduate training in general practice [7]. These centres support trainees through structured seminar programmes, mentoring and participation in regional training networks.

In general, assessments should be objective, valid, reliable [8], [9] and sensitive to the context in which professional performance occurs [3]. Within medical education, assessment is considered a key driver of learning (“assessment drives learning”) [10], [11]. Furthermore, competency-based, practical and standardised assessment formats have gained increasing importance over recent decades [12], [13] and have become well established at many universities. Concepts such as Competency-Based Medical Education (CBME) and Entrustable Professional Activities (EPAs) have moved increasingly into focus [14]. The revised German Model Specialty Training Regulations [1] have likewise adopted a competency-based orientation; however, the format of the specialist examination has remained largely unchanged. From a psychometric perspective, the current specialist examination is insufficient for the valid assessment of competency-based outcomes and practical clinical skills [15]. According to the principle of constructive alignment [16], learning objectives, educational activities and assessment methods should be closely aligned to facilitate goal-directed learning. At present, alignment between the competency objectives of the MWBO and the predominantly unstructured oral examination format appears limited. From an educational assessment perspective, substantial further development of the specialist examination is therefore warranted to achieve better alignment with the competency goals of the revised MWBO. As the final component of postgraduate training in general practice, the specialist examination has important implications for healthcare quality. A recent survey among general practice trainees and specialists found little demand for changes to the current examination format [17]. The aim of this exploratory study was therefore to investigate how examiners evaluate the current examination format, what motivates them to undertake examining activities, which strengths and weaknesses they attribute to the existing assessment, which areas for further development they identify, what qualifications they consider necessary for examiners and to what extent they perceive the Competence Centres for Postgraduate Training to influence examination performance.

## Material and methods

### Study design and setting

A qualitative study using semi-structured interviews with examiners in general practice was conducted. A qualitative approach was chosen to enable an in-depth exploration of experiences and perceived challenges in their complexity and contextual embeddedness. Recruitment, interviews and subsequent data analysis were carried out over a 16-week period between December 2024 and April 2025. Reporting of the study followed the *Consolidated Criteria for Reporting Qualitative Research* (COREQ) guidelines (see attachment 1 ) [18].

### Participants and recruitment

All participants were examiners in general practice and were contacted via email. As no publicly available national register or network of specialist examiners exists in Germany, purposive sampling was employed to ensure variation with regard to federal state, examination experience and gender.

Examiners were recruited both through existing professional networks (Competence centres for postgraduate training and professional conferences) and through the targeted identification of previously unknown individuals based on examination records. Some participants were known to the researchers through professional contacts. However, no hierarchical or other dependent relationship existed between researchers and participants.

A total of 40 examiners were contacted, of whom 13 responded and 12 ultimately participated in an interview.

### Development of the interview guide

Following a literature review (NIH/PubMed^®^ search terms: *“specialist exam”, “board certification”, “resident assessment”* and* “postgraduate medical education assessment”*), an interview guide was developed by a team of four researchers (ACD, CB, MR and SS) with expertise in qualitative research, general practice and medical education.

Kern's six-step approach to curriculum development [19] served as the theoretical framework for structuring the study, as it systematically links needs assessment, goal definition, educational activities and assessment. Both a needs assessment of the target group of examiners and the positioning of the specialist examination within the broader context of postgraduate training were considered during the development of the interview guide.

A semi-structured interview format was chosen to ensure systematic coverage of the key thematic domains while simultaneously allowing for free associations and follow-up questions.

The interview guide was validated using the think-aloud technique [20], in which participants verbalise their cognitive processes, strategies and decision-making while responding to questions. Subsequently, the interview guide was piloted in two interviews and revised slightly (see attachment 2 ). The final interview guide included questions on sociodemographic characteristics, experience as an examiner, perceptions of the current specialist examination, perspectives on potential changes to the examination format and possible effects of the Competence Centres for Postgraduate Training.

### Data collection

Interviews were conducted via Zoom^®^ (San Jose, California, USA; version 6.3.0) by ACD (specialist in psychosomatic medicine and psychotherapy, physician in advanced postgraduate training in general practice, examiner for psychological psychotherapists and child and youth psychotherapists, with several years of experience in conducting interviews and qualitative data analysis).

At the beginning of each interview, participants were informed about the background of the study and the role of the researcher. No third parties (non-participants) were present either at the researcher’s workplace or in the private environments of the participants during the interviews. Audio recordings were made for all interviews. Field notes were taken to document relevant observations made during data collection. Interview transcripts were not returned to participants for commenting or correction.

### Qualitative data analysis

All interviews were transcribed verbatim and analysed manually using a paper-based approach. Transcripts were reviewed and coded using an inductive-deductive qualitative content analysis approach based on Kuckartz [21]. Two researchers (ACD and SS) independently analysed the material. Categories and representative quotations were subsequently compared and revised where necessary. In cases of disagreement or uncertainty, a third researcher (CB) reviewed the material in order to reach consensus.

Throughout the analytical process, continuous reflexivity was maintained regarding personal, interpersonal, methodological and contextual factors that might have influenced data interpretation.

## Results

### Interview characteristics

A total of 12 interviews were conducted between December 2024 and April 2025. The mean interview duration was 33 minutes (range: 24-57 minutes). No technical problems occurred during data collection and none of the interviews had to be repeated.

The coding framework was reviewed after the first two interviews and no further modifications were required. No new themes or codes emerged in the final interviews, indicating that meaning saturation had been achieved and no additional participants were recruited.

### Participant characteristics and examination experience

Participants’ sociodemographic characteristics are presented in table 1 (Tab. 1). All participating examiners were specialists in general practice and possessed substantial expertise in conducting specialist examinations. Across the participating federal states, differences existed regarding whether the chairperson of the examination board was required to belong to the specialty of general practice or another medical discipline. Furthermore, audio recording of examinations was not implemented consistently across federal states.

### Motivation for serving as an examiner

Motivation for undertaking examiner responsibilities were predominantly driven by idealistic and intrinsic factors (see table 2 (Tab. 2)). Financial compensation, which was generally perceived as insufficient to offset lost income, was identified as a potential barrier to recruiting new examiners.

### Strengths and weaknesses of the current examination format

Perceived strengths and weaknesses of the current examination format are summarised in table 3 (Tab. 3). Representative quotations are presented in attachment 3 .

Most examiners identified organisational strengths as well as content-related advantages of the current examination format. A major strength reported by the majority of participants was the feasibility of conducting the examination with relatively little time investment and good practical applicability. Examinations conducted by specialist peers were considered to provide professionally sound quality assurance and to enable the assessment of professional attitudes. Some participants additionally regarded the individual face-to-face examination setting and the flexibility in selecting questions and topics as advantages.

At the same time, several limitations were identified. Most participants criticised the limited objectivity of oral examinations. In particular, the validity of the oral format for assessing practical patient care was questioned, especially in relation to the complexity of long-term care for multimorbid patients in general practice.

*“We only see the idealised picture that the candidate verbally presents to us about how they would manage the cases discussed. We have no opportunity to observe their everyday practice, their actual performance or their doctor–patient communication.” (#1)*

Some participants further identified structural disadvantages, including the late timing of the examination at the end of training, the limited opportunity to influence the educational process, examiner uncertainty and the administrative burden associated with failed examinations.

Within the discussion of strengths and weaknesses, participants emphasised that competencies not directly assessed during the examination may nevertheless be present and that the examination is only of limited suitability for comprehensively assessing competencies within the available timeframe.

*“In my view, it is impossible to pursue too many objectives within 30 minutes. The purpose is for examiners to determine whether the candidate is capable of working independently and unsupervised as a general practitioner in their own practice.” (#2)*

### Criteria for becoming an examiner

All participants reported that they had been appointed by their regional medical association and that, to their knowledge, no transparent selection process existed. Table 4 (Tab. 4) summarises the criteria participants considered important for the selection of examiners.

Several participants questioned whether objective criteria alone would adequately capture personal suitability for the examiner role. One examiner proposed collegial discussions based on case analyses as a potential approach for assessing both professional and personal competencies of prospective examiners.

### Perspectives on future developments

Participants expressed differing views regarding the need for changes to the current examination format (see table 5 (Tab. 5)). Some considered the status quo satisfactory and opposed what they described as “schoolification” of specialist examinations according to university assessment principles.

The majority, however, favoured optimisation and further development of the existing format. In particular, participants advocated the introduction of shared assessment frameworks and predefined expectations in order to improve the examination process. Some additionally proposed supplementing the current examination with written and practical assessment components.

Three participants expressed support for longitudinal assessment models based on international examples, arguing that such approaches would better capture developmental processes and competencies over time. One participant suggested that participation in such assessments could be voluntary.

Perceptions of the potential impact of changes to the examination on postgraduate training varied. While some participants anticipated little effect, others considered examination reform to offer considerable opportunities for improving training quality.

*“In my opinion, changing the examination could make a substantial contribution to improving both the quality of and satisfaction with postgraduate training. If assessment became broader, trainees would have to adapt, learn differently and place different demands on their trainers.” (#10)*

Overall, most participants agreed that the primary emphasis should be placed on improving the quality of postgraduate training rather than creating increasingly difficult examinations.

### Perceived effects of the competence centres for postgraduate training

Approximately half of the interviewed examiners were aware whether candidates had participated in a Competence Centre for Postgraduate Training, for example through active enquiry or prior acquaintance from seminars and were therefore able to comment on possible effects.

These participants reported that trainees who had attended a CCPT appeared to perform better in examinations and demonstrated greater methodological competence and more structured approaches in answering questions. They also attributed higher levels of practical clinical competence to these trainees.

At the same time, participants acknowledged that distinguishing the specific effects of CCPT participation from other influencing factors was difficult. For example, diverse training experiences involving several ambulatory rotations were considered an important predictor of examination performance. Examiners also emphasised that non-participants could perform equally well in specialist examinations.

Nevertheless, a commonly perceived trend was that most candidates who failed the specialist examination had not participated in a CCPT programme.

*“What we can say is that those who participated in a competence centre generally perform very well in the examinations. This does not mean that someone who did not attend a competence centre cannot be excellent, but those who performed poorly were usually not affiliated with a competence centre.” (#11)*

## Discussion

This qualitative study explored examiners' perspectives on the current specialist examination in general practice, including its perceived strengths and weaknesses, potential areas for further development and the influence of competence centres for postgraduate training. In addition, the study investigated examiners’ motivations for participating in assessment activities and their views on the qualifications required for examiners.

The demographic changes within the medical workforce [22] are also reflected among specialist examiners. Given the increasing number of specialist certifications in general practice, a growing need for the recruitment and qualification of new examiners can be anticipated. Participants primarily reported intrinsic and discipline-related motivations for serving as examiners. Adequate financial compensation for lost income may facilitate the recruitment of additional examiners.

The findings reveal an ambivalent attitude towards the current specialist examination. Although participants agreed that the examination enables them to determine whether candidates meet the minimum standard required for specialist certification, they simultaneously acknowledged substantial limitations regarding objectivity, reliability, validity and contextual sensitivity. These findings are consistent with international research on oral examinations. In their review, Memon et al. [23] demonstrated that oral examinations remain deeply embedded within postgraduate medical training in many countries, although some countries have replaced them with alternative formats because of their limited psychometric properties. In Switzerland, for example, specialist certification in general internal medicine includes a written examination consisting of 120 multiple-choice questions, which increases assessment objectivity [24]. Burch et al. [25] questioned the extent to which specialist certification examinations can serve as internationally comparable indicators of physician competence. According to Miller’s pyramid [15], oral examinations predominantly assess cognitive knowledge and clinical reasoning (“knows” and “knows how”), whereas higher levels of competence relating to actual clinical performance (“shows how” and “does”) cannot be adequately captured in an oral format alone. However, the assessment of independent professional practice represents the central purpose of specialist certification as the culmination of postgraduate training. Participants expressed highly heterogeneous views regarding possible examination reforms. Overall, there appeared to be limited motivation among examiners to fundamentally change the current assessment format. Similarly, Fink et al. [17] reported little interest among trainees in general practice in modifying the existing examination. At present, there appears to be no substantial bottom-up initiative for reform, either among examiners or among physicians in postgraduate training. Nevertheless, participants identified several short-term and easy to implement measures for improving the current examination format, including examiner training, regular exchange among examiners (e.g. examiner quality circles) and the development of jointly agreed assessment frameworks and expected standards. Such measures may improve administration objectivity, scoring objectivity and interpretative objectivity. Beckers et al. [26] demonstrated considerable variation in examination content and competency levels in specialist examinations in internal medicine and recommended the implementation of examination blueprints similar to those used in the United States to ensure comparable examination conditions for all candidates. In several European countries, examiners are already required to participate in formal examiner training before conducting assessments [27]. Examiner training programmes implemented in Southern Baden have shown high levels of acceptance among participants [28]. Examiners considered the definition of minimum standards and harmonisation of expected performance levels and assessment criteria essential for improving examination validity and reliability.

Based on the qualification criteria identified by the examiners in the study, it is possible to derive a profile of graduates of examiner training programs, which can serve as the basis for further developing corresponding teaching and learning objectives for such training programs. Examiner training should address both practical competencies in conducting assessments and knowledge of different assessment formats, while simultaneously providing opportunities for critical reflection on the balance between practical feasibility and educational assessment standards. The active involvement of examiners will be essential for any sustainable restructuring of the examination system.

Analogous to the requirement for newly appointed postgraduate trainers in Germany to attend train-the-trainer courses [29], mandatory compact examiner training programmes for newly appointed examiners could be considered. Such programmes are already established in countries including Denmark, the Netherlands and the United Kingdom [27]. Targeted training may also reduce uncertainty and administrative burden in cases of examination failure, while predefined assessment frameworks may provide a transparent and defensible basis for pass-fail decisions. The 2018 German model specialty training regulations (MWBO) describe primarily formal aspects of specialist examinations [1]. Given the transition toward a competency-based approach in the new model speciality training regulations, it would be beneficial to align the examination format accordingly with this competency-based approach. Proposals for structured oral examinations aimed at improving quality and standardisation have already been published [30]. From an educational perspective, formative assessments (“assessment for learning”) are better suited to supporting continuous learning processes than summative examinations (“assessment of learning”). In psychotherapy training in Germany, for example, there is an intermediate examination and a final examination. The final examination consists of a written multiple-choice test and an oral case study examination [https://www.gesetze-im-internet.de/psychthappro/BJNR044800020.html]. Internationally, formative assessment approaches have become established in postgraduate training in general practice in countries such as Denmark and the Netherlands [31], [32], where summative final examinations are largely absent. In France, trainees document their competency development in portfolios that are supervised by educational mentors (*référents pédagogiques*) [33]. Similarly, Khan et al. [34] demonstrated advantages of formative assessment for enhancing individual learning trajectories in postgraduate urology training. Competencies should therefore be continuously assessed and supported through formative workplace-based assessment methods, such as Mini Clinical Evaluation Exercises (Mini-CEX), Direct Observation of Procedural Skills (DOPS) or portfolio assessments [35]. In recent years, Entrustable Professional Activities (EPAs) have emerged as promising instruments for capturing higher levels of clinical performance within Miller’s framework [36]. However, evidence from the Netherlands suggests that EPAs may currently be more valuable as learning tools than as high-stakes assessment instruments in general practice training [37]. Thiessen et al. [38] proposed a best-practice model for specialist examinations based on the triangulation of multiple assessment methods, blueprinting, predefined questions and expected standards, quality assurance procedures and educational impact on candidates. Such approaches inevitably require greater financial and personnel resources [27].

Overall, the findings reveal a tension between awareness of the shortcomings of the current examination format and motivation for fundamental reform. From an assessment-theoretical perspective, further development appears advisable in order to achieve better alignment between competency goals and assessment methods. Given the importance of assessment both for learning processes and for quality assurance in healthcare, such adaptation appears educationally necessary. At the same time, participants repeatedly emphasised practical implementation challenges, particularly the limited availability of examiners and concerns regarding feasibility.

A longitudinal formative assessment programme based on portfolios and logbooks, including practical assessment components, could also strengthen the quality and efficiency of postgraduate training in Germany. Although elements of this are currently addressed through educational supervisors and logbooks, an independent assessment body responsible for triangulating competence assessments may provide additional quality assurance. As many candidates enter general practice training through career transitions or lateral entry [39], formative assessments could provide early feedback regarding their professional reorientation and guide subsequent learning goals. International examples further demonstrate the value of progress testing in postgraduate education [40], enabling trainees to monitor learning progress and identify future educational needs. Participation in formative assessments for feedback purposes could therefore also be considered on a voluntary basis.

A proportion of participants attributed better examination performance and lower failure rates to trainees who had participated in CCPT programmes. These observations may be interpreted as potential effects of the Competence Centres for Postgraduate Training. However, selection effects involving particularly motivated trainees, as well as other confounding factors such as stronger professional networks, must also be considered. Particularly noteworthy was the frequently reported observation that candidates who failed the examination had often not participated in CCPT programmes.

### Strengths and limitations

This study included examiners with both extensive and limited examination experience, thereby strengthening the credibility and richness of the findings. Nevertheless, several limitations should be acknowledged. As a qualitative study, the findings do not claim completeness or representativeness. Examiners from all German federal states were not included and the total number of general practice specialist examiners in Germany is currently unknown. However, the sample included both less experienced and highly experienced examiners and is likely to reflect the composition of examination committees reasonably well. A potential selection bias must be considered, as recruitment was partly conducted through existing professional networks and predominantly attracted already well-connected examiners. It is conceivable that examiners with stronger academic affiliations may be more receptive to innovative assessment formats. In addition, the possibility of social desirability bias within the interviews cannot be excluded. 

The reported effects of the competence centres for postgraduate training should be interpreted as exploratory, as not all participants were aware of candidates’ participation in these programmes and therefore we may not have been able to explore all relevant perspectives. Future mixed-methods studies should further investigate both the feasibility and educational value of potential reforms to the specialist examination as well as the effects of competence centres for postgraduate training.

## Conclusions

Examiners demonstrated considerable awareness of the limitations of the current specialist examination and critically reflected on its objectivity, reliability and validity in relation to the complexity of general practice. Although views regarding desirable reforms varied, participants considered the current examination capable of assessing the minimum standard required for specialist certification.

From an educational perspective, clearer specification of the objectives of the specialist examination, improved alignment between the competency goals of the model specialty training regulations and assessment formats and the introduction of longitudinal assessment approaches incorporating practical assessment components should be considered. As immediately feasible measures, nationwide examiner training, regular exchange among examiners and the implementation of shared assessment frameworks appear particularly promising.

## Notes

### Ethics approval and consent to participate

The study adhered to the Declaration of Helsinki. All participants gave written consent to participate in the study and to the pseudonymised use of their data. The studies were approved by University of Heidelberg, Medical University, Ethic Committee Alte Glockengießerei 11/169115 (S711/2023). The studies were conducted in accordance with the local legislation and institutional requirements. The participants provided their written informed consent to participate in this study. 

### Availability of data and materials

The datasets presented in this article are not readily available because as required by the European Data Protection law the participants were informed of the privacy policy and agreed that the pseudonymized data would be available to the project team and would be stored at the study center. Request to access the datasets should be directed to Simon Schwill (simon.schwill@med.uni-heidelberg.de). 

### Funding

The authors declare that financial support was received for the research, authorship, and/or publication of this article. The KWBW Verbundweiterbildungplus^®^ is supported by public funding under Section 75a of the German Social Code V, Annex 

### Author contributions

ACD was integrated in idea, in conceptualisation and methodology, to acquisition, analysis and interpretation of data and writing the original draft. CB was integrated into acquisition, analysis and interpretation of data. MR was integrated in methodology, to analysis of data. AA was integrated to interpretation of data and reviewing the manuscript. SS was integrated in idea and funding for the study, in conceptualisation and methodology, to analysis and interpretation of data and writing the original draft.

### Authors’ ORCIDs

- Andreas Christian Dreher: [0009-0003-6688-8807]
- Christine Becker: [0009-0009-4802-5761]
- Marco Roos: [0000-0003-1596-5908]
- Attila Altiner: [https://orcid.org/0000-0002-2429-933X]
- Simon Schwill: [0000-0002-0954-2194]

## Acknowledgements

We like to thank all participating GP examiners, all KWBW practices and partner for their participation. The study was presented at the annual conference of the DACH society for medical education in the health professions, Düsseldorf 2025. 

## Competing interests

ACD, CB and SS declare to be integrated in the organisation of the postgraduate training programme KWBW. All authors declare that they have no competing interests.

## Supplementary Material

COREQ (COnsolidated criteria for REporting Qualitative research) checklist

Specialist examination/effects of the competency-based curriculum (KW) – specialist examiners

Illustrative quotes

## Figures and Tables

**Table 1:**
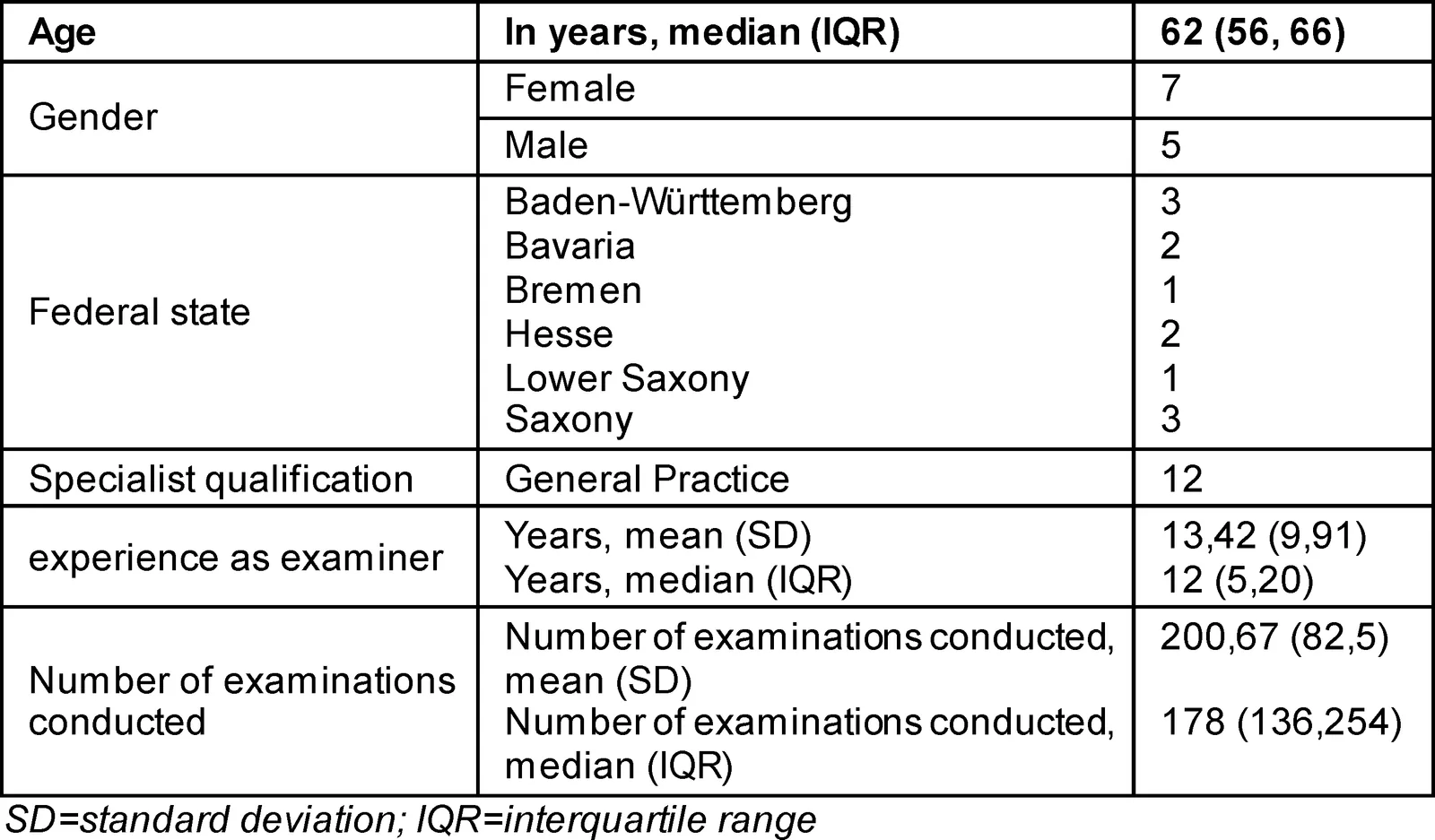
Sociodemographic characteristics of participating specialist examiners in general practice (N=12)

**Table 2:**
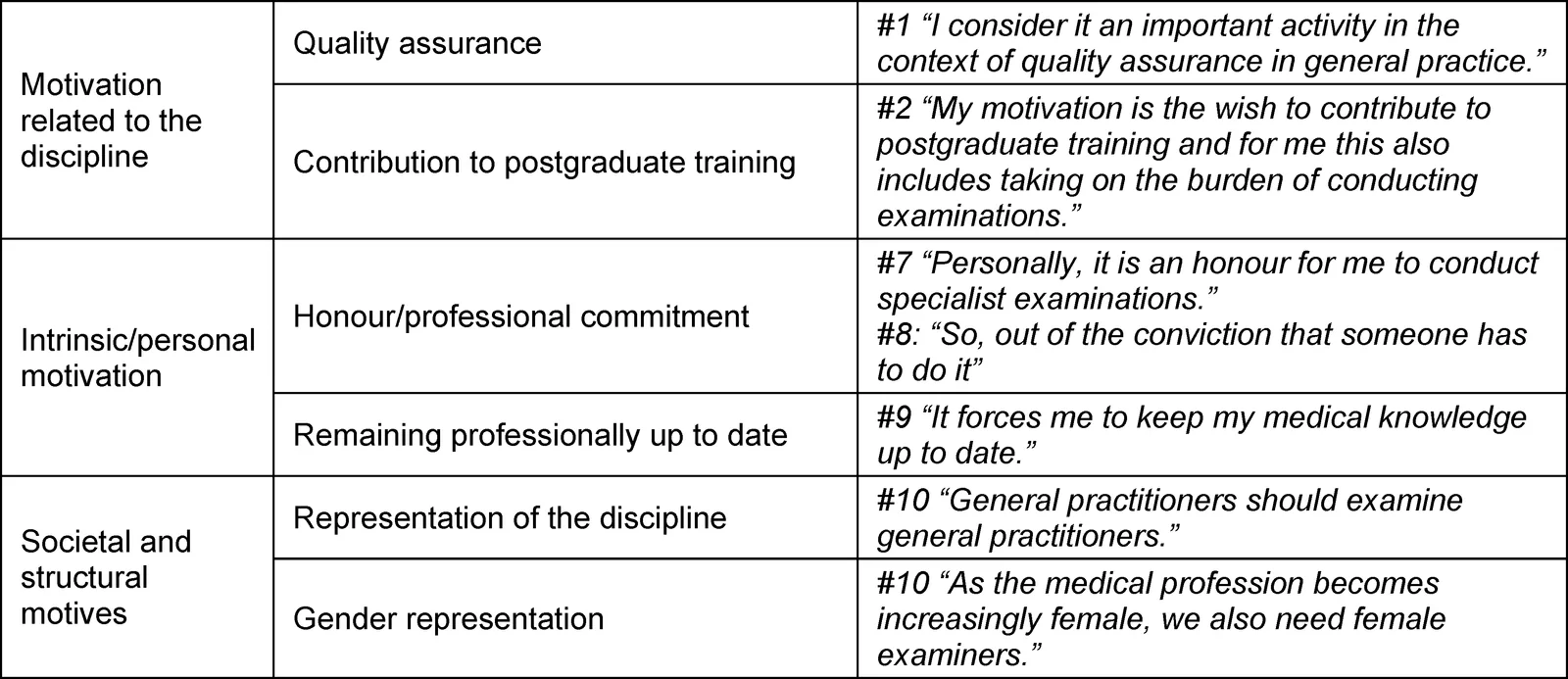
Reasons for serving as a specialist board examiner

**Table 3:**
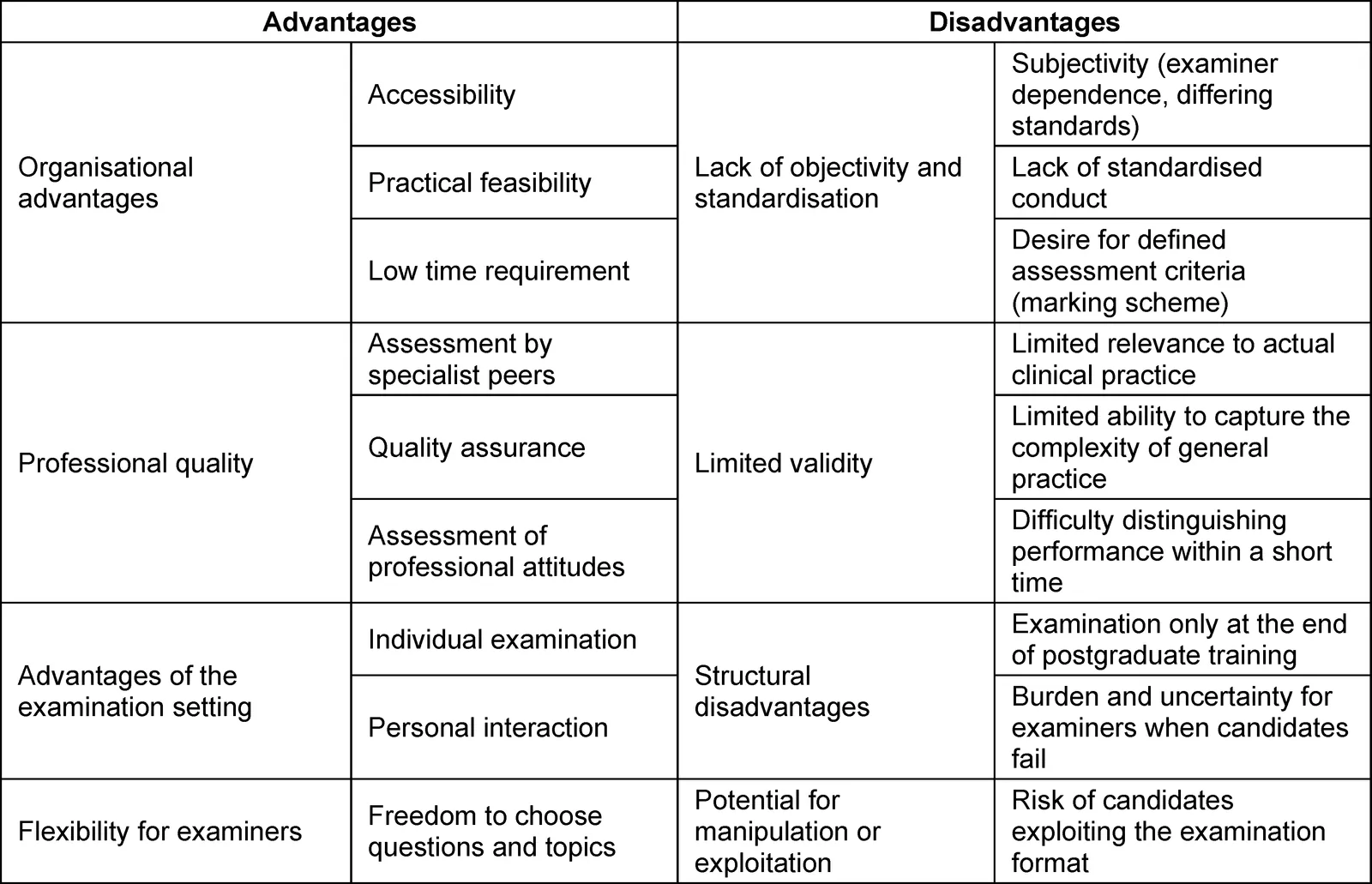
Advantages and disadvantages of the current specialist examination format as perceived by examiners

**Table 4:**
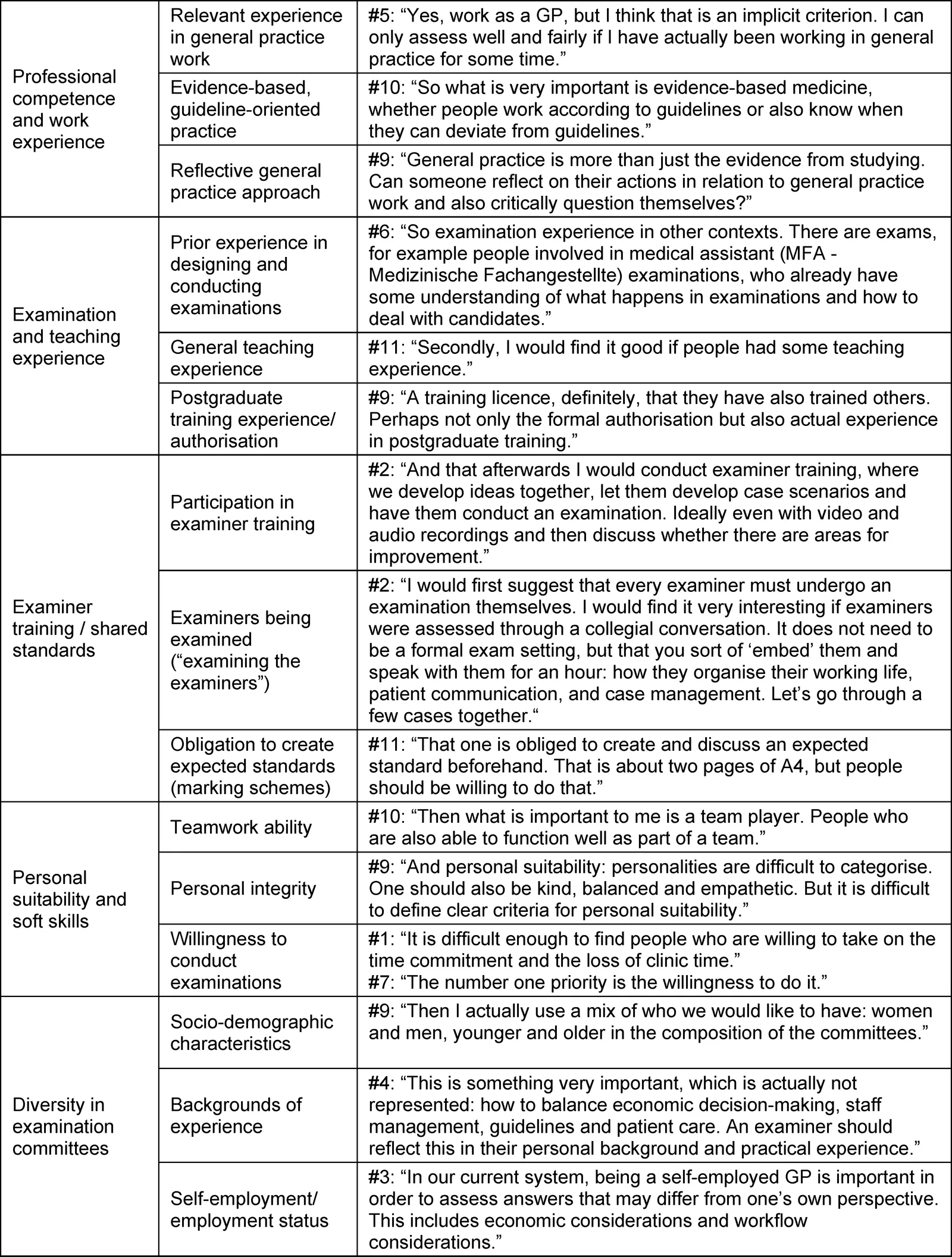
Criteria identified for examiners in general practice from the examiners’ perspective

**Table 5:**
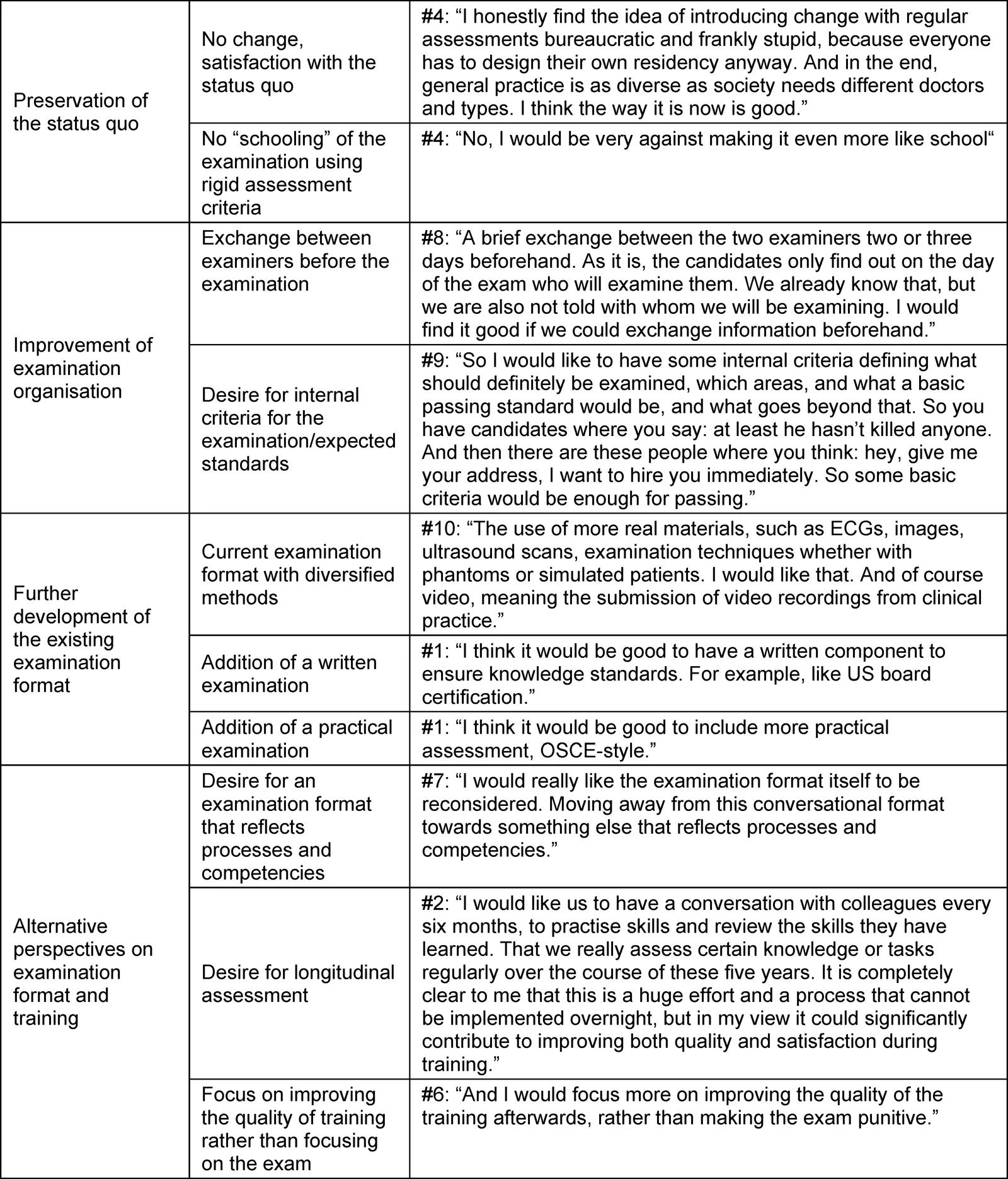
Specialist examiners’ perspectives on changes to the current examination format
