# Supplementary material for: The general practice specialist examination – examiners’ perspectives on the status quo and future development: An exploratory qualitative study
Source: GMS J Med Educ. 2026 Sep 15;43(6):Doc78. doi: 10.3205/zma001872 (PMC13617336; doi:10.3205/zma001872)
Supplement: Supplementary file 1 [file JME-43-78-s-001.pdf]

Attachment 1: COREQ (COnsolidated criteria for REporting Qualitative research) Checklist

A checklist of items that should be included in reports of qualitative research. You must report the page number in your manuscript where you consider each of the items listed in this checklist. If you have not included this information, either revise your manuscript accordingly before submitting or note N/A.

Topic	Item No.	Guide Questions/Description	Reported on Page No.
**Domain 1: Research team and reflexivity**			
Personal characteristics			
Interviewer/facilitator	1	Which author/s conducted the interview or focus group?	3, data collection
Credentials	2	What were the researcher's credentials? E.g. PhD, MD	3, data collection
Occupation	3	What was their occupation at the time of the study?	3, data collection
Gender	4	Was the researcher male or female?	3, data collection
Experience and training	5	What experience or training did the researcher have?	3, data collection
Relationship with participants			
Relationship established	6	Was a relationship established prior to study commencement?	3, data collection; 2, participants and recruitment
Participant knowledge of the interviewer	7	What did the participants know about the researcher? e.g. personal goals, reasons for doing the research	3, data collection; 2, participants and recruitment
Interviewer characteristics	8	What characteristics were reported about the interviewer/facilitator? e.g. Bias, assumptions, reasons and interests in the research topic	3, data collection
**Domain 2: Study design**			
Theoretical framework			
Methodological orientation and Theory	9	What methodological orientation was stated to underpin the study? e.g. grounded theory, discourse analysis, ethnography, phenomenology, content analysis	3, qualitative data analysis
Participant selection			
Sampling	10	How were participants selected? e.g. purposive, convenience, consecutive, snowball	2, participants and recruitment
Method of approach	11	How were participants approached? e.g. face-to-face, telephone, mail, email	2, participants and recruitment
Sample size	12	How many participants were in the study?	3, interview characteristics
Non-participation	13	How many people refused to participate or dropped out? Reasons?	2, participants and recruitment

Setting			
Setting of data collection	14	Where was the data collected? e.g. home, clinic, workplace	3, data collection
Presence of non-participants	15	Was anyone else present besides the participants and researchers?	3, data collection
Description of sample	16	What are the important characteristics of the sample? e.g. demographic data, date	2, participants and recruitment; 3 participants characteristics; 4 table 1 sociodemographics
Data collection			
Interview guide	17	Were questions, prompts, guides provided by the authors? Was it pilot tested?	3, development of the interview guide
Repeat interviews	18	Were repeat inter views carried out? If yes, how many?	None, 3, interview characteristics
Audio/visual recording	19	Did the research use audio or visual recording to collect the data?	3, data collection
Field notes	20	Were field notes made during and/or after the interview or focus group?	3, data collection
Duration	21	What was the duration of the interviews or focus group?	3, interview characteristics
Data saturation	22	Was data saturation discussed?	3, interview characteristics
Transcripts returned	23	Were transcripts returned to participants for comment and/or correction?	3, data collection
**Domain 3: Analysis and findings**			
Data analysis			
Number of data coders	24	How many data coders coded the data?	3, qualitative data analysis
Description of the coding tree	25	Did authors provide a description of the coding tree?	3, qualitative data analysis
Derivation of themes	26	Were themes identified in advance or derived from the data?	3, qualitative data analysis
Software	27	What software, if applicable, was used to manage the data?	none
Participant checking	28	Did participants provide feedback on the findings?	No, 3, data collection
Reporting			
Quotations presented	29	Were participant quotations presented to illustrate the themes/findings? Was each quotation identified? e.g. participant number	table 3-5
Data and findings consistent	30	Was there consistency between the data presented and the findings?	4-7, results
Clarity of major themes	31	Were major themes clearly presented in the findings?	4-7, results
Clarity of minor themes	32	Is there a description of diverse cases or discussion of minor themes?	5-7, results

Developed from: Tong A, Sainsbury P, Craig J. Consolidated criteria for reporting qualitative research (COREQ): a 32-item checklist for interviews and focus groups. *International Journal for Quality in Health Care*. 2007. Volume 19, Number 6: pp. 349 – 357

Once you have completed this checklist, please save a copy and upload it as part of your submission. DO NOT include this checklist as part of the main manuscript document. It must be uploaded as a separate file.

Attachment 1 to Dreher AC, Becker C, Ross M, Altiner A, Schwill S. *The general practice specialist examination – examiners' perspectives on the status quo and future development: An exploratory qualitative study.* GMS J Med Educ. 2026;43(6):Doc78. DOI: 10.3205/zma001872
