# Supplementary material for: The general practice specialist examination – examiners’ perspectives on the status quo and future development: An exploratory qualitative study
Source: GMS J Med Educ. 2026 Sep 15;43(6):Doc78. doi: 10.3205/zma001872 (PMC13617336; doi:10.3205/zma001872)
Supplement: Supplementary file 2 [file JME-43-78-s-002.pdf]

Attachment 2: Specialist examination/effects of the competency-based curriculum (KW) – Specialist examiners

**Date / No.:**

INTERVIEW GUIDE – INDIVIDUAL INTERVIEWS

How is the status quo of the specialist examination assessed by examiners and what changes with an impact on postgraduate training are conceivable?<br>What intended and unintended effects of the competency-based curriculum (KW) have already been achieved or could be expected in the future?

1) **Consent: Has information been provided and has written informed consent been obtained?**

2) **Before the interview begins, we ask for the following socio-demographic data:**

age, gender/pronouns	
Profession	Year of specialist certification/graduation from medical school
Working environment/ activity	Currently working as a GP?<br>Training supervisor?<br>Since when examiner? Approximately how many examinations conducted?<br>Examination remuneration (in euros)<br>Proportion of candidates passed/failed (%)
Specialist examination	What are the framework conditions for the specialist examination in your federal state?<br>• How is the examination conducted? Please describe.<br>• Duration, number of examiners, documentation/protocol<br>• How is performance reflected and assessed? (Which categories exist? Pass/fail, internal grading systems?)<br>• How is the examination result communicated?

3) **So now we would start the recording**

**Topic**	**Question**	**Time**
Consent<br>Thank you very much for agreeing to participate in this interview. Before recording, I would like to confirm again that you have received the information sheet, that all your questions about the study have been answered and that you consent to video and audio recording of the interview. Please answer yes or no..		1 min<br><br>**START**
**Introduction/ personal motivation**	The specialist examination takes place after at least 6 years of study and 5 years of full-time equivalent postgraduate training.<br>**What personally motivates you to work as an examiner for the specialist qualification in general practice?**	2 Min
**Aim of the specialist examination**	**In your opinion, what is the aim of the examination? (open question)**	3 Min
**Status quo**<br><br>**Advantages/ disadvantages**	**How satisfied are you with the current format of the specialist examination?**<br>➢ Where do you see advantages of the current format?<br>➢ Where do you see disadvantages?<br>➢ (If applicable: Has anything changed over time in the examination format?)	5 Min
**Training report**	**How relevant are the training documentation and the training report for you?**	2 Min
**Quality criteria of the examination**	**Imagine you had to develop a measurement method for the specialist examination, what criteria would you want to assess?**	2 Min

**Desired changes**	**Would you like to change anything about the examination?**<br>If yes, what?<br>➢ If we think further in theoretical terms, what additional changes would you like?	3 Min
**Selection of examiners**	**How did you become a specialist examiner?**<br>**How are examiners recruited in your experience?**<br>➢ According to which criteria are they selected?	2 Min
**Prior examination experience**	**Did you already have examination experience beforehand?**<br>**Do you also conduct examinations in other contexts?**	2 Min
**Reflection on examination competence**	**Are there collegial exchange opportunities accessible to you regarding examination activities?**<br>➢ Do you engage in regular training in assessment/examination?<br>➢ What training opportunities for examiners are you aware of?<br>➢ What experiences have you had with them?	2 Min
**Personal learning curve**	How would you describe your personal learning curve as an examiner?	2 Min
**Qualification of examiners**	**How are or should** *(ideally)* **examiners for specialist examinations be qualified in your opinion?**<br>**If you had to develop criteria for examiners, what would they be?**	2 Min
**Impact of the examination on postgraduate training**	*Assuming the examination were changed in the future:* **How do you assess the likelihood that such a change would influence postgraduate training?**	2 Min
**Effects of in the examination**	Apart from the specialist examination itself, are you aware of which doctors have participated in a competency-based curriculum (KW)?<br>• If yes, do you see differences? Which ones?<br>• If no, I would ask more generally about possible effects of KW	4 Min
**General effects of KW**	Participation in a KW for doctors in training in general practice is now established in all federal states. Do you perceive any effects? If so, which? How do you identify them?	3 Min
**Unintended positive effects of KW**<br>**Unintended negative effects**	The KW were introduced to improve the quality and efficiency of postgraduate training. Beyond this, do you perceive other effects, possibly surprising ones that go beyond the original objectives?<br>If applicable: Are there also negative effects? If yes, for whom?	5 Min
**Future unintended effects of KW**	Speculating: what further effects of KW can you imagine for the future (next 10 years)?	3 Min
**Coming to the end**	Thank you very much for your support in our study. Looking back at the interview: has everything relevant about the specialist examination and the effects of KW been covered in your opinion? Would you like to add anything?	2 Min
**End**	We will now end the interview recording. Thank you very much!	

Attachment 2 to Dreher AC, Becker C, Ross M, Altiner A, Schwill S. *The general practice specialist examination – examiners' perspectives on the status quo and future development: An exploratory qualitative study.* GMS J Med Educ. 2026;43(6):Doc78. DOI: 10.3205/zma001872
