# Supplementary material for: The general practice specialist examination – examiners’ perspectives on the status quo and future development: An exploratory qualitative study
Source: GMS J Med Educ. 2026 Sep 15;43(6):Doc78. doi: 10.3205/zma001872 (PMC13617336; doi:10.3205/zma001872)
Supplement: Supplementary file 3 [file JME-43-78-s-003.pdf]

Attachment 3: Illustrative quotes

1a. Advantages of the current specialist examination format as perceived by examiners

**Advantages**		Illustrative quote
Organisational advantages	Accessibility	***#7:*** *"(One advantage is that) it is easily accessible. For candidates with language barriers or examination anxiety, this format may be more acceptable."*
	Practical feasibility	***#8:*** *"Because it is also a practical matter. When there are many candidates, they all have to be examined. From a pragmatic perspective, the process simply has to be manageable. We have to be able to get through all the examinations."*
	Low time requirement	***#1:*** *"Short and usually painless."*
Professional quality	Assessment by specialist peers	***#6:*** *"As specialists in family medicine, we are the ones who determine whether someone is suitable for the profession. This is not done by people from other specialties; we are the ones making that decision."*
	Quality assurance	***#1:*** *"I consider it an important activity in terms of quality assurance in family medicine. (…) It is sufficient as a quality control measure in the sense that it filters out the most obvious failures. The most obvious ones."*
	Assessment of professional attitudes	***#12:*** *"In contrast, an oral examination allows professional attitudes to be assessed well."*
Advantages of the examination setting	Individual examination	***#6:*** *"It is an individual examination. Previously, examinations were conducted in groups of three."*
	Personal interaction	***#7:*** *"You can engage in a conversation and get an impression of how the other person thinks (…). At most, I can gain an impression of what kind of person they are and, perhaps, how much knowledge they have."*
Flexibility for examiners	Freedom to choose questions and topics	***#4:*** *"I am relatively free to decide what I want to assess. That means I can emphasize the aspects that I consider important instead of being forced to cover things that I do not consider essential. That is definitely an advantage."*

Attachment 3 to Dreher AC, Becker C, Ross M, Altiner A, Schwill S. *The general practice specialist examination – examiners' perspectives on the status quo and future development: An exploratory qualitative study.* GMS J Med Educ. 2026;43(6):Doc78. DOI: 10.3205/zma001872

1b. Disadvantages of the current specialist examination format as perceived by examiners

Disadvantages		Illustrative quote
Lack of objectivity and standardisation	Subjectivity (examiner dependence, differing standards)	***#10:*** *"A fundamental disadvantage is that candidates may encounter idiosyncratic examiners. I think that is a real risk."*<br>***#7:*** *"Yes, (a disadvantage is) that it is highly subjective—very much influenced by subjectivity."*
	Lack of standardised conduct	***#9:*** *"However, it is not really standardized. It is largely left to the individual examiner—what they ask and how they ask it—which can be problematic."*
	Desire for defined assessment criteria (marking scheme)	***#9:*** *"I would like to have a set of internal criteria specifying what should definitely be assessed, which domains should be covered, what constitutes the minimum standard for passing, and what goes beyond that."*
Limited validity	Limited relevance to actual clinical practice	***#1:*** *"All we see is the idealized version that the candidate describes verbally—what they claim they would do in the presented cases. We have no opportunity to observe their everyday practice, their actual performance, or their doctor–patient communication."*
	Limited ability to capture the complexity of general practice	***#8:*** *"After all, we want to assess family medicine. To me, family medicine involves dealing with highly complex clinical problems. And that is exactly where the limited time and examination format become problematic."*
	Difficulty distinguishing performance within a short time	***#4:*** *"Can you really differentiate? Well, is the candidate simply anxious, or do they actually lack knowledge? Are they just good at talking, or is there real substance behind it—or not? In a 30-minute conversation, it is actually quite difficult to determine that."*
Structural disadvantages	Examination only at the end of postgraduate training	***#9:*** *"It takes place very late, at the end of training, so we have very little opportunity to provide feedback to candidates. Something like a preliminary examination would make sense."*
	Burden and uncertainty for examiners when candidates fail	***#8:*** *"You are simply uncertain, and it is not pleasant to be the person who has to fail someone."*
Potential for manipulation or exploitation	Risk of candidates exploiting the examination format	***#10:*** *"I have encountered several colleagues where I had the impression that they passed on the basis of general intelligence rather than because they were well prepared."*
